# Sacralization of Coccygeal Vertebra: A Descriptive Observational Study in Bangladesh

**DOI:** 10.7759/cureus.27496

**Published:** 2022-07-31

**Authors:** Rawshon Ara Naznin, Md Moniruzzaman, Sharmin Akter Sumi, Maskura Benzir, Iffat Jahan, Rahnuma Ahmad, Mainul Haque

**Affiliations:** 1 Anatomy, Thengamara Mohila Sabuj Sangha (TMSS) Medical College, Bogra, BGD; 2 Pediatric Surgery, 250 Bedded General Hospital, Kushtia, BGD; 3 Anatomy, Bangabandhu Sheikh Mujib Medical University (BSMMU), Dhaka, BGD; 4 Physiology, Eastern Medical College, Bangladesh, Cumilla, BGD; 5 Physiology, Medical College for Women and Hospital, Dhaka, BGD; 6 Pharmacology and Therapeutics, National Defence University of Malaysia, Kuala Lumpur, MYS

**Keywords:** perineal tear, prolonged labor, coccydynia, sacral foramina, anomaly, lumbosacral transitional vertebrae, sacral vertebrae

## Abstract

Background: In the sacrococcygeal region, anatomical variation is due to the sacralization of the coccygeal vertebra, which is the due union of/fusion of the fifth sacral with the first coccygeal vertebra of five couples of sacral foramina under-detected or asymptomatic beyond radiological assessment. That is why it is challenging to know the cause of coccydynia, caudal block failure, the difficult second stage of labor, and perineal tears. The present study aims to improve knowledge about the anatomical variation of sacralization of the coccygeal vertebra. Additionally, to find the prevalence of sacralization of coccygeal vertebra in Sylhet, Bangladesh.

Methods: This study was performed on 60 parched, totally calcified, typical sacra of mature-age individuals of undetermined sexes, fulfilling the inclusion criteria from the bone bank of the osteology museum of the Department of Anatomy, Sylhet MAG Osmani Medical College, Sylhet, Bangladesh, from July 2017 to June 2018. Sex determination of the collected unknown sacra was conducted using discriminant function analysis. It was found that 50% (30) were male and 50% (30%) were female. The unpaired *t*-tests and chi-square were utilized to determine the statistical significance.

Results: Out of 60 sacra, eight (13.33%) samples presented with sacralization. This study found that males had significantly higher straight (p=0.05) and curved (p=0.05) lengths of sacrococcygeal vertebrae. The sacrococcygeal curvature index (SCI) showed statistically significant (p=0.05) differences between the sexes.

Conclusion: Sacralization may exert an impact on the caudal block. It could extend the second stage of the labor process with perineal tears. Therefore, knowledge about the anatomical variation of the coccygeal vertebra is essential.

## Introduction

The typical vertebral column consists of seven cervicals, twelve thoracics, five lumbar, five sacral, and four coccygeal vertebrae, thereby totaling thirty-three [1]. Multiple studies reported that the disparities in the vertebral column occur from the typical configuration [2-4]. Furthermore, lumbosacral transitional vertebrae (LSTV) are inborn spinal paradoxes through either sacralization (bottommost lumbar segment) or lumbarization (topmost sacral part) of the vertebral column [5]. LSTV is a frequent but incidental detection among the populace [6], with a reported prevalence of 4-36% [6-8]. The sacrum is a considerable triagonal bone positioned at the bottom of the backbone [9,10]. It helps transmit body mass load from the chest and abdomen to the pelvis and lower extremities [9,10]. The sacrum is self-possessed by a union of five vertebrae to configure a wedge-shaped bony cornucopia with four twosomes of sacral openings [10]. The typical sacrum has four pairs of frontward (pelvic) and rearward (dorsal) sacral orifices, which give passage to the sacral canal and corresponding sacral nerves that travel by virtue of these spaces (between sacral bone [SB] 1 and SB2 [S1 space], SB2 and SB3 [S2 space], SB3 and SB4 [S3 space], SB4 and SB5 [S4 space]) [11,12]. The existence of five duos of the sacral opening is an anatomical abnormality formed because of the attachment of an additional vertebra at the crown or rear end of the sacrum [11]. The combination of the fifth lumbar with the first sacral bone resulted in the sacralization of the lumbar vertebrae [10,13]. It is often described as the ossification of the first coccygeal bone with the fifth sacral or the peak of the sacrum, known as the sacralization of the coccygeal vertebra at the caudal end [13]. The reported prevalence of sacralization varies from 4.1-11% [7,14].

The five-pair sacral opening builds as a result of coccygeal sacralization, and the fifth pair of sacral and coccygeal nerves travel through this opening [15]. Sacralization infrequently causes symptoms and may remain unnoticeable throughout life [16,17]. Often, sacralization is diagnosed through a roentgenogram (X-Ray) examination for other pathological issues [17]. The detailed knowledge of sacral anatomical divergences impacts different medical arenas (orthopedic surgeons, neurosurgeons, neurologists, urologists, anesthesiologists, obstetricians, radiologists, forensic doctors, and all surgical specialists operating close to the vertebral column) [18-20]. So, precise knowledge of sacralization is vital to avoid complications among patients with bony anomalies [21,22].

Embryology of sacralization process

People are often born with sacralization [7,23]. Vertebrae are derived from the sclerotome portion of the somites [7,24,25]. *Hox 5* genes regulate the modeling of the distinctive architecture of the vertebral column [26,27]. *Hox 5* genes mutations may perhaps lead to this anomaly [28]. Sacralization of the coccygeal vertebra with the peak of the sacrum is instigated by higher expression of *Hox 11* genes in the somite phase [26,27,29]. Although genetics may play a vital role, nonetheless [5,30,31], the exact cause of sacralization is yet unknown [13,32]. Less common whys and wherefores could be a catastrophic injury, uttermost arthritic inflammatory modification, and clinically indicated spinal fusion surgery [33-35].

Clinical implication of sacralization

The sacralization of the coccygeal vertebra often promotes coccydynia, failure of caudal anesthesia, the troublesome and extended second phase of labor, and perineal laceration [9,36]. Patients with a fused coccyx who could not flex while sitting were more likely to experience coccygeal pain than those with a normal coccyx [37,38]. The sacrococcygeal joint has been wiped out by the fusion of the first coccygeal segment with the sacrum [39,40]. It has been reported that there is a strong relationship between sacralization and low back pain (LBP) [13,41,42]. Sacralization is not continually interrelated to LBP; it can remain symptom-free for eras [16,43,44]. The pain due to the sacralization process is usually slow onset. It progressively changes to dreadful, possibly because of compulsion on nerve/nerve trunks, ligamentous strain, compression of soft tissues between bony joints, the concurrent existence of arthritis, or bursitis [33].

Sacralization and Caudal Nerve Block

The sacrum is an indispensable anatomical structure for the caudal epidural block. This anesthetic block is frequently used to diagnose and treat lumbar spine illnesses [45,46]. The caudal anesthetic procedure is frequently required for multiple surgical needs such as herniorrhaphy, surgery of the lower extremities, lower abdominal surgery (cesarean section, prostate surgery), etc. [47]. Sacralization makes it problematic to spot the landmark need for caudal epidural block, leading to caudal block failure [47]. The caudal procedure is also used in pediatric cases for postoperative analgesia [48]. Consequently, there may be insufficient analgesia due to sacral anatomical anomalies [49].

Sacralization and Obstetrics

Usually, the first coccygeal vertebra is mobile and is pushed posteriorly during the second phase of labor [15]. Therefore, the anteroposterior diameter of the pelvis outlet expands and facilitates giving birth [15]. The coccyx becomes fixed when fused with the sacrum [39,50]. Consequently, the anteroposterior outlet diameter does not increase [51,52], which may lead to protracted labor and perineal injuries [53,54]. Sacralization also causes trouble during the instrumental parturition process. Subsequently, evaluating all pregnant women for sacralization is highly recommended to ensure better clinical outcomes [20,55].

Nonetheless, this is a frequently encountered anatomical and clinical anomaly of the sacrum. A few or less number of research studies have been conducted in Bangladesh and several low- and middle-income countries (LMICs; underdeveloped and developing countries). The knowledge of anatomy and anatomical variation [56-58] of the sacrococcygeal region is fundamental in many clinical situations, including the most vital issue of normal vaginal childbirth.

Objectives of the study

The current research is intended to determine the anatomical variations (sacralization of the coccygeal vertebra (incidence/prevalence) of the sacrum and coccyx through morphometric assessments among the Bangladeshi community. Therefore, this study possibly helps Bangladeshi populations and other geopolitical arenas effectively treat these anatomical ailments. This study's statistics can be used to compare similar research findings locally and overseas. Hopefully, our data will enhance the database of anatomical variants of the sacrum.

## Materials and methods

Study details

Study Design and Sample Selection

This was a descriptive, non-experimental investigation study. The research samples were collected from the Department of Anatomy, Sylhet MAG Osmani Medical College, Sylhet. Sacra from both men and women were incorporated for this research. The sex was grouped by discriminant function analysis. The sacrum was scrutinized to evaluate its vertebral components, and the number of sacral openings was counted. Non-sacralization was viewed as the sacrum showing 04 pairs of orifices and 05 vertebral sections. Alternatively, sacralization was considered as the sacrum showed 05 pairs of openings and 06 vertebral parts.

Study Period, Sampling Method, and Sample Size

This research was conducted from July 2017 to June 2018. Consecutive opportunity sampling was applied to choose the sacrum, and the sample size was 60.

Data Collection Tool and the Procedure of Data Collection

**Figure 1:**
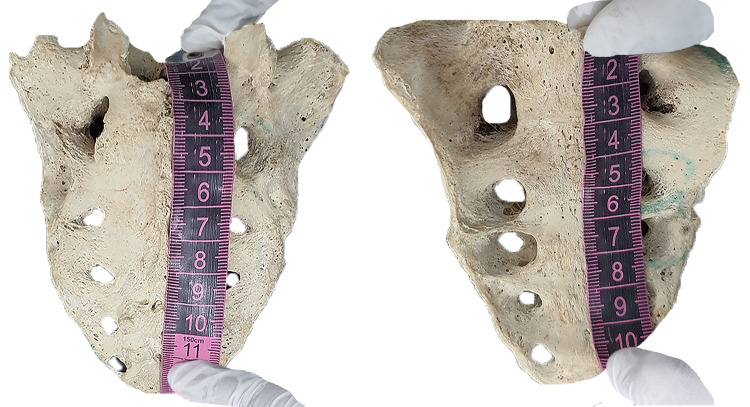
Illustrating measurements of Sacralized bone on ventral and dorsal surfaces utilizing measuring tape.

**Figure 2:**
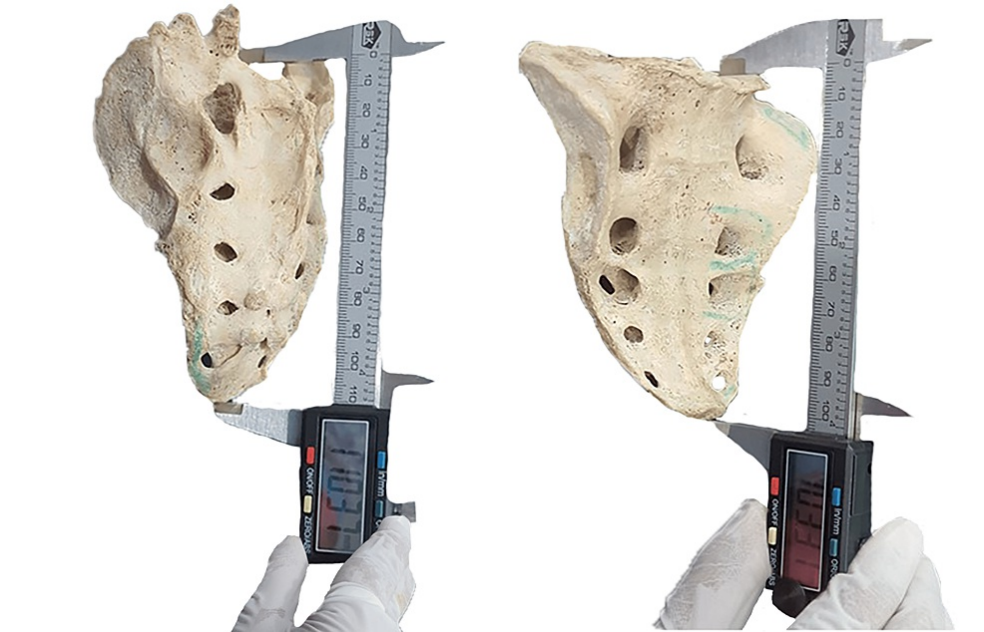
Illustrating measurements of sacralized bone on both ventral and dorsal surfaces utilizing digital slide calipers.

Measuring tape (Figure 1) and digital slide calipers (Figure 2) are the tools used for data collection. Data are collected with the help of digital slide calipers along the mid-line of the sacrum from the middle of the anterosuperior margin of the promontory to the middle of the anteroinferior margin of the last coccygeal vertebra. It was recorded in millimeters, and the curved length of the sacrococcygeal vertebra was recorded using flexible ribbon tape. The current study also determines the sacrococcygeal curvature index (SCI). SCI is assessed through "sacrococcygeal straight length divided by sacrococcygeal curved length × 100" [59].

Data Analysis and Interpretation

Data were processed manually and analyzed with the help of Statistical Package for Social Sciences (SPSS) Version 22.0. (IBM Corp. Released 2013, IBM SPSS Statistics for Windows, Version 22.0. Armonk, NY: IBM Corp.).

## Results

**Figure 3:**
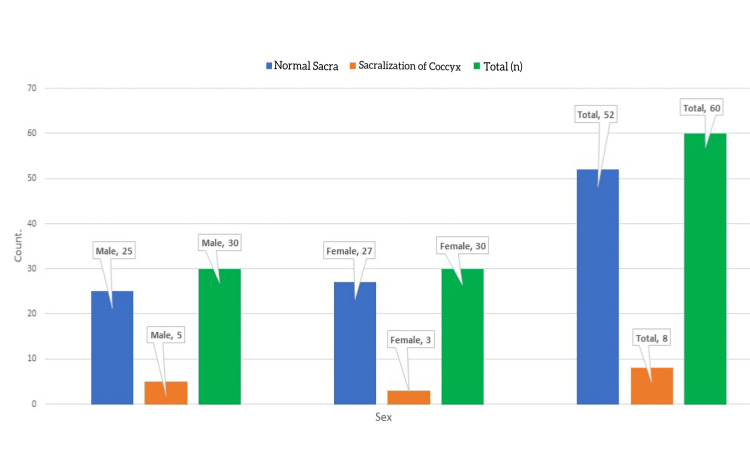
Bar Diagram Showing the Frequency Distribution of Sacralization of the Coccygeal Vertebra.

**Figure 4:**
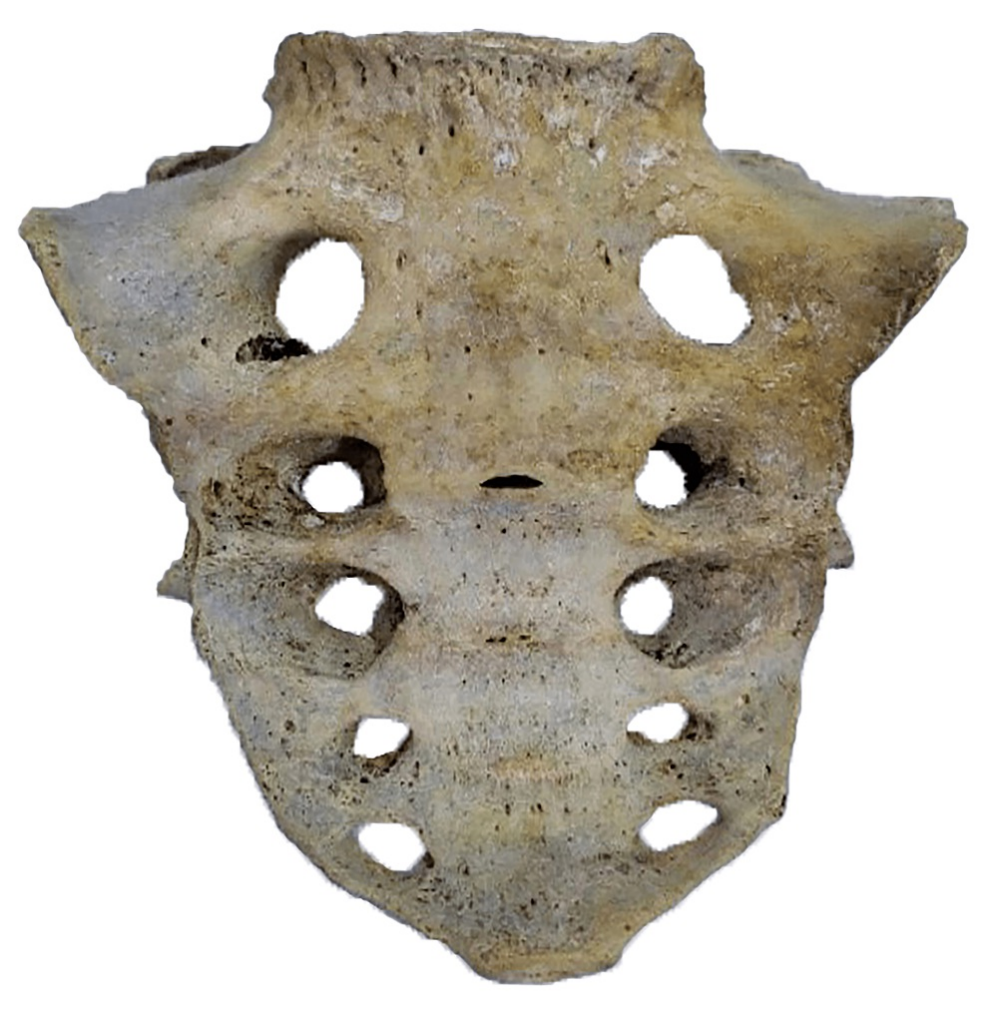
Illustrating Ventral Surface of Sacrum With 05 Pairs of Sacral Foramina.

**Figure 5:**
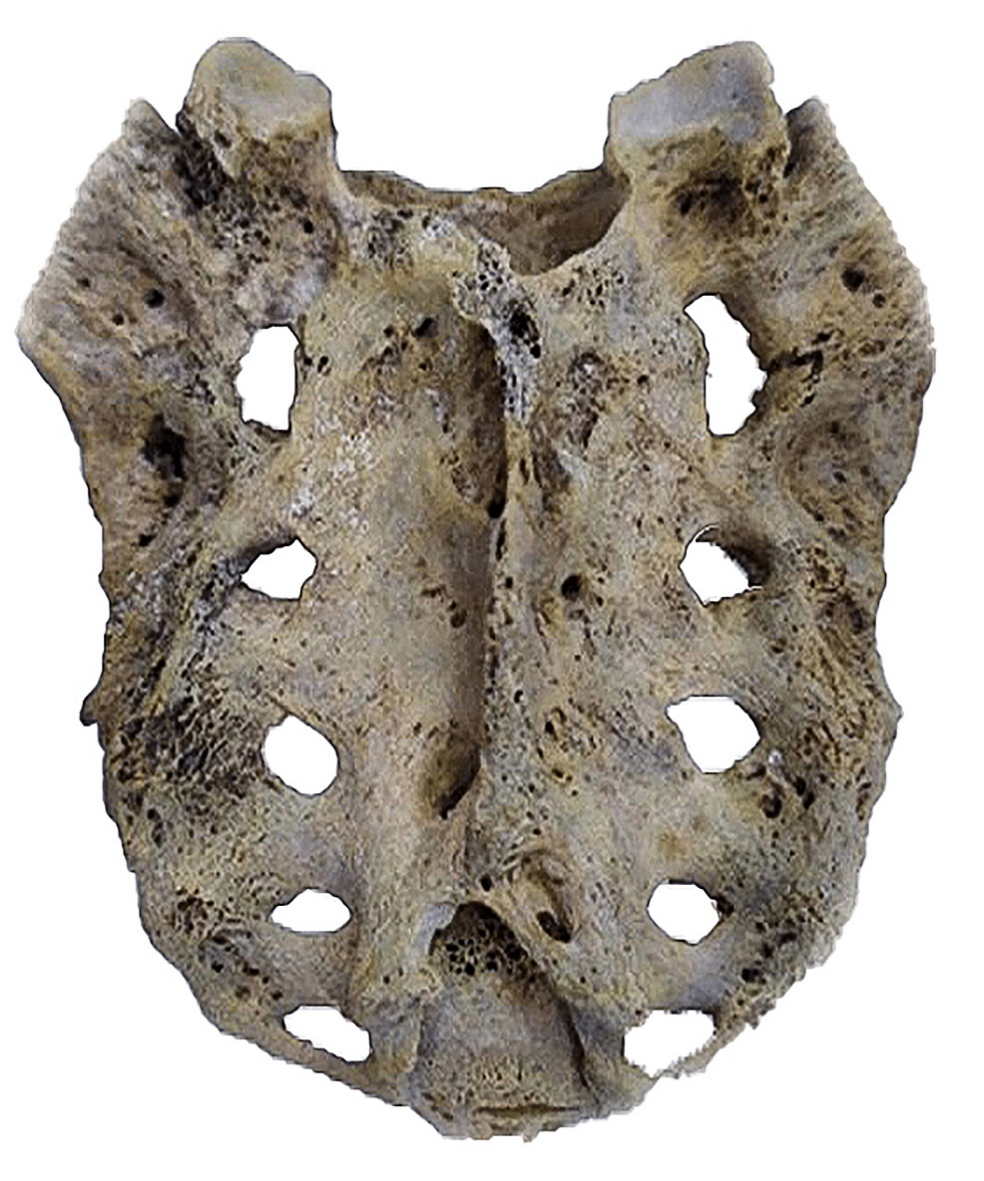
Illustrating Dorsal Surface of Sacrum With 05 Pairs of Sacral Foramina.

Among the study sample, 86.67% (fifty-two) of sacra were typical. Only 13.33% (eight) sacra showed sacralization of the coccygeal vertebra. Table 1 shows the sacralization of the coccygeal vertebra. This study revealed that males (8.33%) had a higher prevalence of sacralization than females (5%). Figure 3 shows the frequency distribution of sacralization of the coccygeal vertebra. Figures 4-5 show the sacrum (ventral surface and dorsal surface) with 05 pairs of sacral orifices. There was no statistically significant (p=0.706) correlation between sex and sacralization (Table 2). Among the present study population, males had statistically significantly longer straight (p=0.05) and curved (p=0.05) lengths of sacrococcygeal vertebrae (Table 3). In this study, population males possess statistically significantly (p=0.05) higher SCI (Table 4).

**Table 1:** Frequency Distribution of Sacralization of the Coccyx.

Sacra	Male	%	Female	%	Percent (%)
Normal sacra	25	41.67%	27	45%	52 (86.67%)
Sacralization of coccyx	5	8.33%	3	5%	8 (13.33%)
Total (n)	30	50%	30	50%	60 (100%)

**Table 2:** Association of Sacralization of Coccyx Between Sex. Note: Chi-square-test was conducted to determine statistical significance between sex. n: number of the samples, ns: not significant (p>0.05).

	Sex		Total	Chi-square	p-value
	Male	Female			
Normal Sacra	25 (83.3%)	27 (90.0%)	52	0.577	0.706^ns^
Sacralization of Coccyx	5 (16.7%)	3 (10.0%)	8		
	100.0%	100.0%	60		

**Table 3:** Comparison of Straight Length and Curve Length of Sacrococcygeal Vertebrae Between Male and Female. Note: Data expressed as mean ± SD, n: number of the samples, statistical analysis done by Student’s unpaired t-test; *statistically significant test (p<0.05).

Variable	Sex	Measurement (mm)		P-value
		Range	Mean ± SD	
Straight length	Male (5)	101–130	110.8±11.7	0.05*
	Female (3)	65–71	68±3	
Curve length	Male (5)	119–132	125.2±6.3	0.05^*^
	Female (3)	82–108	96.67±13.32	

**Table 4:** Comparison of Sacrococcygeal Curvature Index Between Male and Female. Note: Data expressed as mean ± SD, n: number of the samples, statistical analysis conducted by Student’s unpaired t-test; *statistically significant test (p<0.05).

Variable	Sex	Measurement (mm)			P-value
		Range	Index average	Mean ± SD	
Sacrococcygeal curvature index	Male (5)	101–130	88.52	88.52±8.13	0.05^*^
	Female (3)	65–71	71.59	71.59±13.56	

## Discussion

The global existence of LSTV has been reported to be 4-36% [60]. The LSTV is comprised of sacralization or lumbarization [5,7]. Sacralization is a congenital vertebral abnormality of the lumbosacral spine that often leads to improper recognition and difficulty spotting a vertebral component [13]. Therefore, changes in the biomechanical anatomy of the vertebral column and related anatomical body constituents lead to important clinical consequences, especially in anesthetic techniques and surgical procedures [5]. Furthermore, multiple studies have reported sacral malformation as high as 50% [61,62]. Anatomic congenital deformity sacralization was written almost ten decades ago [63,64]. In this study, the sacralization of coccygeal vertebra among the Bangladeshi population was 13.33%. Among Indians, Nepalese, and Israel, sacralization was reported as 6-11.1% [36,65], 11.1% [66], and 13.1% [67], respectively [36,65-67]. One more recent Indian study revealed 17.7% of sacralization [68]. Therefore, the current study findings were in the same line as studies conducted in neighboring countries of Bangladesh. There has been stated that no statistically significant variability was observed in the prevalence of sacralization between sex in this study. Nevertheless, there have been significant (p<0.001) and non-significant (p>0.05) differences observed in the earlier surveys among the Turkish and Israeli populations, respectively [68,69]. Consequently, our findings were similar [68] and dissimilar [69] to earlier studies. This study revealed that both straight and curved lengths of sacrococcygeal vertebrae were statistically significantly (p=0.05) higher among males. Similar findings were statistically significant in earlier French [7] and Korean [70] studies. One study was conducted in New Zealand. Similarly, female sacrococcygeal vertebrae are short [3], which promotes coccydynia [71,72]. The word coccydynia was first developed in 1859 by Simpson and is also known as coccygodynia and tailbone pain [37,72]. Later, Foye identified coccygodynia as one of the top reasons for LBP [73,74]. LBP is frequently considered a mysterious disorder as several disease pathologies can cause similar painful conditions. It is often difficult to diagnose the root cause of these disorders [75-77]. Multiple studies were conducted on slum dweller women (82%), bank staff (36.6%), and physiotherapists (60%) who suffer from low back pain [78-80]. One study revealed that a successful coccygectomy had been performed among a group of 20 patients when these patients were resistant to medical therapy [81]. Consequently, this study advocated that health professionals, especially those involved with the childbirth process, should know that sacralization is vital for proper and quick management of this anatomical derivation. Maternal death and prolonged labor are the third leading causes of maternal mortality in Bangladesh and are responsible for 20% of overall demises amongst females [82-85]. It has been reported that around 60-75% of childbirths happen to be non-institutional and, on most occasions, without help from trained birth attendants [86,87]. Sacralization in women reduces the pelvic passage [15,29], causing inconsistency with the fetal head and often leading to prolonged or obstructed labor for several days. The pressure generated over the perineal area causes necrosis that leads to genital fistula in several poor obstetrics healthcare centers, especially in LMICs [88]. Perineal injury (53-89%) is an enormously common and predictable difficulty of normal vaginal delivery. Moreover, sacralization potentiates the issue by reducing the pelvic outlet [89,90]. With Bangladesh being an LMIC, it has been reported that perineal laceration is common in the country [91-96]. The current study findings are significant because sacralization reduces pelvic outlet and increases maternal morbidity. SCI was statistically significantly higher in the present study. It was similar [97] and dissimilar [3] to earlier studies conducted in Kuwait and New Zealand. One study reported that the SCI was statistically significantly nether among coccydynia cases than in the controls and between sex [59]. It has been reported that Bangladeshi women also have coccydynia cases [81]. Multiple overseas studies revealed that coccydynia is predominantly found among female and obese patients [98-100]. Consequently, the present study's findings go along with those of the overseas researchers.

Limitations of this study

This study was cross-sectional research. Thereby containing its inherent limitation that cannot measure appropriate disease frequency, especially for the sporadic disease condition. Cross-sectional research only generates a snapshot of the whole story's total picture, not a video or cinema. Consequently, the study results cannot be generalized to the whole country [101]. Moreover, this single-center study often lacks the scientific consistency or relevant extraneous plausibility required for precise information for extensive utilization of our data [102].

## Conclusions

Knowing about the sacralization of coccygeal vertebrae is very important for radiologists, neurologists, anesthesiologists, pediatric surgeons, obstetricians, and orthopedic surgeons to manage clinical situations such as prolonged labor, perineal tears, regional block, and LBP. This study highlights the depth of knowledge about anatomical variation, which is helpful for effectively managing several medical and health needs. Furthermore, radiological assessment before any interventional spinal anesthesia is critical, especially for pregnancy, childbirth, and lower abdominal surgical procedures related to the sacrococcygeal region. Although the study has many limitations, nevertheless, the data generated through this may possibly serve as baseline information for future research. Further studies regarding the sacralization of the coccygeal vertebra of the human dry sacrum involving multiple centers and with a larger sample size are recommended. Critical research is needed for detailed information on this variant.

## References

[1] DeSai C, Reddy V, Agarwal A (2022). Anatomy, Back, Vertebral Column. https://www.ncbi.nlm.nih.gov/books/NBK525969/.

[2] Chaturvedi A, Klionsky NB, Nadarajah U, Chaturvedi A, Meyers SP (2018). Malformed vertebrae: a clinical and imaging review. Insights Imaging.

[3] Woon JT, Perumal V, Maigne JY, Stringer MD (2013). CT morphology and morphometry of the normal adult coccyx. Eur Spine J.

[4] Indiran V, Sivakumar V, Maduraimuthu P (2017). Coccygeal morphology on multislice computed tomography in a tertiary hospital in India. Asian Spine J.

[5] Jancuska JM, Spivak JM, Bendo JA (2015). A review of symptomatic lumbosacral transitional vertebrae: Bartolutti's syndrome. Int J Spine Surg.

[6] Hanhivaara J, Määttä JH, Niinimäki J, Nevalainen MT (2020). Lumbosacral transitional vertebrae are associated with lumbar degeneration: retrospective evaluation of 3855 consecutive abdominal CT scans. Eur Radiol.

[7] French HD, Somasundaram AJ, Schaefer NR, Laherty RW (2014). Lumbosacral transitional vertebrae and its prevalence in the Australian population. Global Spine J.

[8] de Bruin F, Ter Horst S, Bloem JL (2017). Prevalence and clinical significance of lumbosacral transitional vertebra (LSTV) in a young back pain population with suspected axial spondyloarthritis: results of the SPondyloArthritis Caught Early (SPACE) cohort. Skeletal Radiol.

[9] Nastoulis E, Karakasi MV, Pavlidis P, Thomaidis V, Fiska A (2019). Anatomy and clinical significance of sacral variations: a systematic review. Folia Morphol (Warsz).

[10] Sattar MH, Guthrie ST (2022). Anatomy, Back, Sacral Vertebrae. https://www.ncbi.nlm.nih.gov/books/NBK551653/.

[11] Kapetanakis S, Gkasdaris G, Pavlidis P, Givissis P (2018). Concurrent lumbosacral and sacrococcygeal fusion: a rare aetiology of low back pain and coccygodynia?. Folia Morphol (Warsz).

[12] Diel J, Ortiz O, Losada RA, Price DB, Hayt MW, Katz DS (2001). The sacrum: pathologic spectrum, multimodality imaging, and subspecialty approach. Radiographics.

[13] Bulut M, Uçar BY, Uçar D (2013). Is sacralization really a cause of low back pain?. ISRN Orthop.

[14] Sekharappa V, Amritanand R, Krishnan V, David KS (2014). Lumbosacral transition vertebra: prevalence and its significance. Asian Spine J.

[15] Singh R (2014). Classification and analysis of fifth pair of sacral foramina in Indian dry sacra. Int J Morphol.

[16] Alonzo F, Cobar A, Cahueque M, Prieto JA (2018). Bertolotti's syndrome: an underdiagnosed cause for lower back pain. J Surg Case Rep.

[17] Kumar J, Ali S, Zadran N, Singh M, Ahmed Z (2020). A rare case of Bartolutti's syndrome in a young patient: a case report and literature review. Cureus.

[18] Shah M, Halalmeh DR, Sandio A, Tubbs RS, Moisi MD (2020). Anatomical variations that can lead to spine surgery at the wrong level: part III lumbosacral spine. Cureus.

[19] Shah M, Halalmeh DR, Sandio A, Tubbs RS, Moisi MD (2020). Anatomical variations that can lead to spine surgery at the wrong level: part II thoracic spine. Cureus.

[20] Shah M, Halalmeh DR, Sandio A, Tubbs RS, Moisi MD (2020). Anatomical variations that can lead to spine surgery at the wrong level: part I, cervical spine. Cureus.

[21] Ravikanth R, Majumdar P (2019). Bertolotti's syndrome in low-backache population: classification and imaging findings. Ci Ji Yi Xue Za Zhi.

[22] Tatara Y, Niimura T, Sekiya T, Mihara H (2021). Changes in lumbosacral anatomy and vertebral numbering in patients with thoracolumbar and/or lumbosacral transitional vertebrae. JB JS Open Access.

[23] Ward E. From somites to vertebral column. Ph.D (2022). From somites to vertebral column. University College London, Gower St, London WC1E 6BT, United Kingdom.

[24] Gilbert SF (2022). Paraxial Mesoderm: The Somites and Their Derivatives. https://www.ncbi.nlm.nih.gov/books/NBK10085/.

[25] Hughes DS, Keynes RJ, Tannahill D (2009). Extensive molecular differences between anterior- and posterior-half-sclerotomes underlie somite polarity and spinal nerve segmentation. BMC Dev Biol.

[26] Mallo M, Wellik DM, Deschamps J (2010). Hox genes and regional patterning of the vertebrate body plan. Dev Biol.

[27] Carapuço M, Nóvoa A, Bobola N, Mallo M (2005). Hox genes specify vertebral types in the presomitic mesoderm. Genes Dev.

[28] Quinonez SC, Innis JW (2014). Human HOX gene disorders. Mol Genet Metab.

[29] Singh R (2012). Analytical view of the simultaneous occurrence of sacralization and congenital anomalies. Eur J Anat.

[30] Matson DM, Maccormick LM, Sembrano JN, Polly DW (2020). Sacral dysmorphism and lumbosacral transitional vertebrae (LSTV) review. Int J Spine Surg.

[31] Muir JM (2012). Partial lumbosacral transitional vertebrae: 2 cases of unilateral sacralization. J Chiropr Med.

[32] Drew R, Kjellström A (2022). Sacralization of the fifth lumbar vertebra: under-reported and misunderstood. Zenodo.

[33] Kamal AM, Ara S, Begum S, Hoque MM, Khatun K (2014). Sacralization: sacrum with five pairs of sacral foramina. Bang J Ana.

[34] Yoshihara H (2012). Sacroiliac joint pain after lumbar/lumbosacral fusion: current knowledge. Eur Spine J.

[35] Lee CS, Hwang CJ, Lee SW, Ahn YJ, Kim YT, Lee DH, Lee MY (2009). Risk factors for adjacent segment disease after lumbar fusion. Eur Spine J.

[36] Krishnamurthy A, Adibatti M (2016). Study on incidence of sacralization of fifth lumbar vertebra in South Indian population. Ital J Anat Embryol.

[37] Mabrouk A, Alloush A, Foye P (2022). Coccyx Pain. https://www.ncbi.nlm.nih.gov/books/NBK563139/.

[38] Marinko LN, Pecci M (2014). Clinical decision making for the evaluation and management of coccydynia: 2 case reports. J Orthop Sports Phys Ther.

[39] Tague RG (2011). Fusion of coccyx to sacrum in humans: prevalence, correlates, and effect on pelvic size, with obstetrical and evolutionary implications. Am J Phys Anthropol.

[40] Tetiker H, Koşar MI, Çullu N, Canbek U, Otağ I, Taştemur Y (2017). MRI-based detailed evaluation of the anatomy of the human coccyx among Turkish adults. Niger J Clin Pract.

[41] Gopalan B, Yerramshetty JS (2018). Lumbosacral transitional vertebra-related low back pain: resolving the controversy. Asian Spine J.

[42] Hanhivaara J, Määttä JH, Karppinen J, Niinimäki J, Nevalainen MT (2022). The association of lumbosacral transitional vertebrae with low back pain and lumbar degenerative findings in MRI: a large cohort study. Spine (Phila Pa 1976).

[43] Saha N, Das S, Momin AD (2015). Unilateral Sacralisation - A Case Report. IOSR J Dent Med Sci.

[44] Drew R, Kjellström A (2021). Sacralization in the Mary Rose and Kronan assemblages: an inconsistently recorded anomaly. Int J Osteoarchaeol.

[45] Kao SC, Lin CS (2017). Caudal epidural block: an updated review of anatomy and techniques. Biomed Res Int.

[46] Cleary M, Keating C, Poynton AR (2011). The flow patterns of caudal epidural in upper lumbar spinal pathology. Eur Spine J.

[47] Sanghvi C, Dua A (2022). Caudal Anesthesia. https://www.ncbi.nlm.nih.gov/books/NBK551.

[48] Wiegele M, Marhofer P, Lönnqvist PA (2019). Caudal epidural blocks in paediatric patients: a review and practical considerations. Br J Anaesth.

[49] Kim SG, Yang JY, Kim DW, Lee YJ (2013). Inadvertent dural puncture during caudal approach by the introducer needle for epidural adhesiolysis caused by anatomical variation. Korean J Pain.

[50] Hekimoglu A, Ergun O (2019). Morphological evaluation of the coccyx with multidetector computed tomography. Surg Radiol Anat.

[51] Eggleton JS, Cunha B (2022). Anatomy, Abdomen and Pelvis, Pelvic Outlet. https://www.ncbi.nlm.nih.gov/books/NBK557602/.

[52] Siccardi MA, Imonugo O, Valle C (2022). Anatomy, Abdomen and Pelvis, Pelvic Inlet. https://www.ncbi.nlm.nih.gov/books/NBK519068/.

[53] Mikolajczyk RT, Zhang J, Troendle J, Chan L (2008). Risk factors for birth canal lacerations in primiparous women. Am J Perinatol.

[54] Memon HU, Handa VL (2013). Vaginal childbirth and pelvic floor disorders. Womens Health (Lond).

[55] FA E (1952). The relationship of the effect and pain of pregnancy to the anatomy of the pelvis. Acta radiol.

[56] Bagoji IB, Bharatha A, Prakash KG, Hadimani GA, Desai V, Bulgoud RS (2020). A morphometric and radiological study of sacral hiatus in human adult sacra and its clinical relevance in caudal epidural anaesthesia. Maedica (Bucur).

[57] Mustafa MS, Mahmoud OM, El Raouf HH, Atef HM (2012). Morphometric study of sacral hiatus in adult human Egyptian sacra: their significance in caudal epidural anesthesia. Saudi J Anaesth.

[58] Bagheri H, Govsa F (2017). Anatomy of the sacral hiatus and its clinical relevance in caudal epidural block. Surg Radiol Anat.

[59] Shams A, Gamal O, Mesregah MK (2021). Sacrococcygeal morphologic and morphometric risk factors for idiopathic coccydynia: a magnetic resonance imaging study. Global Spine J.

[60] Becker L, Schömig F, Haffer H, Ziegeler K, Diekhoff T, Pumberger M (2021). Safe zones for spinopelvic screws in patients with lumbosacral transitional vertebra. Global Spine J.

[61] Wu LP, Li YK, Li YM, Zhang YQ, Zhong SZ (2009). Variable morphology of the sacrum in a Chinese population. Clin Anat.

[62] Gardner MJ, Morshed S, Nork SE, Ricci WM, Chip Routt ML Jr (2010). Quantification of the upper and second sacral segment safe zones in normal and dysmorphic sacra. J Orthop Trauma.

[63] Brailsford JF (1929). Deformities of the lumbosacral region of the spine. Br J Surg.

[64] Hasner E, Jacobsen Hh, Schalimtzek M, Snorrason E (1953). Lumbosacral transitional vertebrae; a clinical and roentgenologic study of 400 cases of low back pain. Acta radiol.

[65] Dharati K, Nagar SK, Ojaswini M, Dipali T, Paras S, Sucheta P (2012). A study of sacralisation of fifth lumbar vertebra in Gujarat. Nat J Med Res.

[66] Khatun S, Shah DK (2021). Lumbosacral transitional vertebrae in patients attending a tertiary care hospital of Nepal. Birat J Health Sci.

[67] Dar G, Peled N (2014). The association between sacralization and spondylolisthesis. Anat Sci Int.

[68] Sabnis A, Nakhate M (2020). A Study of Prevalence of Sacralization of L5 Vertebra. Int J Anat Res.

[69] Uçar D, Uçar BY, Coşar Y (2013). Retrospective cohort study of the prevalence of lumbosacral transitional vertebra in a wide and well-represented population. Arthritis.

[70] Yoon MG, Moon MS, Park BK, Lee H, Kim DH (2016). Analysis of sacrococcygeal morphology in Koreans using computed tomography. Clin Orthop Surg.

[71] Sandrasegaram N, Gupta R, Baloch M (2020). Diagnosis and management of sacrococcygeal pain. BJA Educ.

[72] Lirette LS, Chaiban G, Tolba R, Eissa H (2014). Coccydynia: an overview of the anatomy, etiology, and treatment of coccyx pain. Ochsner J.

[73] Foye PM (2017). Coccydynia: Tailbone pain. Phys Med Rehabil Clin N Am.

[74] Foye PM (2010). Stigma against patients with coccyx pain. Pain Med.

[75] Koes BW, van Tulder MW, Thomas S (2006). Diagnosis and treatment of low back pain. BMJ.

[76] Allegri M, Montella S, Salici F (2016). Mechanisms of low back pain: a guide for diagnosis and therapy. F1000Res.

[77] Wong AY, Karppinen J, Samartzis D (2017). Low back pain in older adults: risk factors, management options and future directions. Scoliosis Spinal Disord.

[78] Barua SK, Sultana N (2015). Prevalence of low back pain among women living in slum areas of Dhaka city. Chattagram Maa-O-Shishu Hospital Med Coll J.

[79] Ali M, Ahsan GU, Hossain A (2020). Prevalence and associated occupational factors of low back pain among the bank employees in Dhaka City. J Occup Health.

[80] Mondal R, Saker RC, Akter S, Banik PC, Baroi SK (2018). Prevalence of low back pain and its associated factors among physiotherapists in Dhaka city of Bangladesh in 2016. JOHE.

[81] Islam MA, Batajoo S, Mahmud MSA, Shrestha M (2018). Coccygectomy can be a option for coccydynia, which is refractory to medical treatment. Bangabandhu Sheikh Mujib Med Univ J.

[82] Head SK, Yount KM, Sibley LM (2011). Delays in recognition of and care-seeking response to prolonged labor in Bangladesh. Soc Sci Med.

[83] Koblinsky M, Chowdhury ME, Moran A, Ronsmans C (2012). Maternal morbidity and disability and their consequences: neglected agenda in maternal health. J Health Popul Nutr.

[84] Islam MA, Chowdhury RI, Chakraborty N, Bari W, Akhter HH (2004). Factors associated with delivery complications in rural Bangladesh. Eur J Contracept Reprod Health Care.

[85] Sikder SS, Labrique AB, Shamim AA (2014). Risk factors for reported obstetric complications and near misses in rural northwest Bangladesh: analysis from a prospective cohort study. BMC Pregnancy Childbirth.

[86] (2022). 2011 Bangladesh Demographic and Health Survey. https://dhsprogram.com/pubs/pdf/fr265/fr265.pdf.

[87] Rahman MA, Rahman MA, Rawal LB (2021). Factors influencing place of delivery: evidence from three south-Asian countries. PLoS One.

[88] Trovik J, Thornhill HF, Kiserud T (2016). Incidence of obstetric fistula in Norway: a population-based prospective cohort study. Acta Obstet Gynecol Scand.

[89] Ramar CN, Grimes WR (2022). Perineal Lacerations. https://www.ncbi.nlm.nih.gov/books/NBK559068/.

[90] Goh R, Goh D, Ellepola H (2018). Perineal tears - a review. Aust J Gen Pract.

[91] Banu MA, Nargis S, Rahman MM, Sina MM, Pervin M, Manjari M (2019). Prediction of perineal tear during childbirth by the assessment of striae gravidarum score. Med Today.

[92] Khanam R, Sultana N, Rubaia A, Ahmed B, Sayeeda N, Mridha T (2014). A friendly approach to repair fourth degree perineal tears. Bang Med J.

[93] Ferdous J, Ahmed A, Dasgupta SK (2012). Occurrence and determinants of postpartum maternal morbidities and disabilities among women in Matlab, Bangladesh. J Health Popul Nutr.

[94] Aguiar M, Farley A, Hope L, Amin A, Shah P, Manaseki-Holland S (2019). Birth-related perineal trauma in low- and middle-income countries: a systematic review and meta-analysis. Matern Child Health J.

[95] Fronczak N, Antelman G, Moran AC, Caulfield LE, Baqui AH (2005). Delivery-related complications and early postpartum morbidity in Dhaka, Bangladesh. Int J Gynaecol Obstet.

[96] Rahman M, Rahman SM, Pervin J, Aktar S, El Arifeen S, Rahman A (2020). Body mass index in early-pregnancy and selected maternal health outcomes: findings from two cohorts in Bangladesh. J Glob Health.

[97] Marwan YA, Al-Saeed OM, Esmaeel AA, Kombar OR, Bendary AM, Azeem ME (2014). Computed tomography-based morphologic and morphometric features of the coccyx among Arab adults. Spine (Phila Pa 1976).

[98] Garg B, Ahuja K (2021). Coccydynia: a comprehensive review on etiology, radiological features and management options. J Clin Orthop Trauma.

[99] Howard PD, Dolan AN, Falco AN, Holland BM, Wilkinson CF, Zink AM (2013). A comparison of conservative interventions and their effectiveness for coccydynia: a systematic review. J Man Manip Ther.

[100] Kodumuri P, Raghuvanshi S, Bommireddy R, Klezl Z (2018). Coccydynia - could age, trauma and body mass index be independent prognostic factors for outcomes of intervention?. Ann R Coll Surg Engl.

[101] Wang X, Cheng Z (2020). Cross-sectional studies: strengths, weaknesses, and recommendations. Chest.

[102] Bellomo R, Warrillow SJ, Reade MC (2009). Why we should be wary of single-center trials. Crit Care Med.

